# The unique structural and biochemical development of single cell C_4_ photosynthesis along longitudinal leaf gradients in *Bienertia sinuspersici* and *Suaeda aralocaspica* (Chenopodiaceae)

**DOI:** 10.1093/jxb/erw082

**Published:** 2016-03-08

**Authors:** Nuria K. Koteyeva, Elena V. Voznesenskaya, James O. Berry, Asaph B. Cousins, Gerald E. Edwards

**Affiliations:** ^1^Laboratory of Anatomy and Morphology, VL Komarov Botanical Institute of Russian Academy of Sciences, St Petersburg, 197376, Russia; ^2^Department of Biological Sciences, State University of New York, Buffalo, NY 14260, USA; ^3^School of Biological Sciences, Washington State University, Pullman, WA 99164-4236, USA

**Keywords:** *Bienertia sinuspersici*, C_4_ plants, CO_2_ compensation point, development, immunolocalization, *in situ* hybridization, leaf anatomy, single cell C_4_ photosynthesis, *Suaeda aralocaspica*, ultrastructure.

## Abstract

Temporal and spatial patterns of photosynthetic enzyme expression and structural maturation of chlorenchyma cells along longitudinal developmental gradients were characterized in young leaves of two single cell C_4_ species, *Bienertia sinuspersici* and *Suaeda aralocaspica*. Both species partition photosynthetic functions between distinct intracellular domains. In the C_4_-C domain, C_4_ acids are formed in the C_4_ cycle during capture of atmospheric CO_2_ by phosphoenolpyruvate carboxylase. In the C_4_-D domain, CO_2_ released in the C_4_ cycle via mitochondrial NAD-malic enzyme is refixed by Rubisco. Despite striking differences in origin and intracellular positioning of domains, these species show strong convergence in C_4_ developmental patterns. Both progress through a gradual developmental transition towards full C_4_ photosynthesis, with an associated increase in levels of photosynthetic enzymes. Analysis of longitudinal sections showed undeveloped domains at the leaf base, with Rubisco *rbc*L mRNA and protein contained within all chloroplasts. The two domains were first distinguishable in chlorenchyma cells at the leaf mid-regions, but still contained structurally similar chloroplasts with equivalent amounts of *rbc*L mRNA and protein; while mitochondria had become confined to just one domain (proto-C_4_-D). The C_4_ state was fully formed towards the leaf tips, Rubisco transcripts and protein were compartmentalized specifically to structurally distinct chloroplasts in the C_4_-D domains indicating selective regulation of Rubisco expression may occur by control of transcription or stability of *rbc*L mRNA. Determination of CO_2_ compensation points showed young leaves were not functionally C_4_, consistent with cytological observations of the developmental progression from C_3_ default to intermediate to C_4_ photosynthesis.

## Highlight

In family Chenopodiaceae, two unique forms of single cell C_4_ photosynthesis with different evolutionary origins show convergence in structural and biochemical expression of C_4_ traits during leaf ontogeny.

## Introduction

Carbon gain through photosynthesis is limited in plants due to high levels of photorespiration, a metabolically wasteful process which is prominent in warm climates and under conditions of reduced water availability. Some plant species have evolved the distinct CO_2_ concentrating mechanism known as C_4_ photosynthesis, which suppresses photorespiration. The C_4_ pathway enhances the photosynthetic capacity of these plants relative to other plants that directly assimilate atmospheric CO_2_ via the C_3_ pathway (Edwards and Walker, 1983; Hatch, 1987). C_4_ photosynthesis has evolved independently more than 60 times across a wide range of monocot and dicot species (Edwards and Voznesenskaya, 2011; Sage *et al.*, 2011). An important requirement for C_4_ function is spatial separation of two biochemical processes: the initial capture of atmospheric CO_2_ during the carboxylation phase of the C_4_ cycle occurs within one compartment (the carboxylation domain, C_4_-C), while the decarboxylation phase donates CO_2_ to ribulose-1,5-bisphosphate carboxylase/oxygenase (Rubisco) within a second compartment (the decarboxylation domain, C_4_-D). There are many structural and biochemical variations associated with anatomical development and function of the two domains (Edwards and Walker, 1983; Hatch, 1987; Edwards and Voznesenskaya, 2011; Sage *et al.*, 2011). In its most common form, this separation is achieved by the dual-cell C_4_ system called Kranz anatomy. The leaves of Kranz species possess two biochemically and anatomically distinct photosynthetic cell types: an inner layer called bundle sheath (BS) or Kranz cells, and an outer layer of palisade mesophyll (M) cells. In the leaves of Kranz C_4_ plants, the C_4_-C domain is specifically compartmentalized to the M cells, while the C_4_-D domain is compartmentalized to BS cells (Edwards and Walker, 1983; Hatch, 1987; Kanai and Edwards, 1999).

Remarkably, a few terrestrial C_4_ species that lack Kranz anatomy have been shown to function by development of C_4_-C and C_4_-D domains within different parts of individual chlorenchyma cells (Edwards *et al.*, 2004; Freitag and Stichler, 2000; Voznesenskaya *et al.*, 2001, 2002; Akhani *et al.*, 2005; Chuong *et al.*, 2006; Edwards and Voznesenskaya, 2011). These unique species occur only in subfamily Suaedoideae (family Chenopodiaceae). Within this subfamily, independent evolutionary acquisition of C_4_ photosynthesis is considered to have occurred four times. This includes two origins of single cell C_4_ (SC-C_4_) with different forms of anatomy in genus *Bienertia* versus *Suaeda aralocaspica* (syn.=*Borszczowia aralocaspica*), as well as two separate origins of distinctive Kranz C_4_ anatomies between *Suaeda* sections *Salsina sensu lato* (*s.l.*) versus *Schoberia* (Schütze *et al.*, 2003; Kapralov *et al.*, 2006). All four lineages utilize a mitochondrial NAD-malic enzyme (NAD-ME) for the decarboxylation phase of the C_4_ cycle in the C_4_-D domain.

There are major structural differences between the two domains of *S. aralocaspica* and *Bienertia* species. In *S. aralocaspica*, biochemically dimorphic chloroplasts are partitioned between opposite ends of the elongated chlorenchyma cells; the distal end towards the exterior is the C_4_-C domain, and the proximal end towards the interior of the leaf is the C_4_-D domain. In contrast, in *Bienertia* species the dimorphic chloroplasts are partitioned between the peripheral cytoplasm (the peripheral compartment, PC) that functions as the C_4_-C domain and a ball-like central cytoplasmic compartment (CCC) in the center of the cell that functions as the C_4_-D domain, with the two compartments connected by cytoplasmic channels through the vacuole (Voznesenskaya *et al.*, 2001, 2002; Edwards and Voznesenskaya, 2011).

In all forms of C_4_ that have been studied to date, during leaf development a progression occurs in structural and biochemical differentiation, which results in fully functional C_4_ photosynthesis. To understand the process of C_4_ development, it is essential to identify the regulatory processes and factors responsible for these transitional steps. Precise methods are needed for defining the sequence of these transitions and exactly where they occur in different types of C_4_. In Kranz-type C_4_ species, this is being approached through analyses of changes during development in mesophyll and bundle sheath cells along leaf longitudinal gradients to ultimately identify genetic components of C_4_ form and function; for a review, see Covshoff *et al.* (2014). This includes studies in representatives from families Poaceae (maize and *Arundinella hirta*), Cyperaceae (three anatomical types), Amaranthaceae (*Amaranthus hypochondriacus*), Chenopodiaceae (*Suaeda taxifolia, S. eltonica*) and Cleomaceae (*Cleome angustifolia, Gynandropsis gynandra*), which show some differences in development (Langdale *et al.*, 1988; Wang *et al.*, 1992; Dengler *et al.*, 1995; Soros and Dengler, 2001; Wakayama *et al.*, 2003; Li *et al.*, 2010; Majeran *et al.*, 2010; Koteyeva *et al.*, 2011, 2014; Nelson, 2011; Pick *et al.*, 2011; Aubry *et al.*, 2014; Külahoglu *et al.*, 2014).

In the current work, an analogous approach was used to analyze chlorenchyma development along longitudinal leaf gradients in two species, *Bienertia sinuspersici* and *Suaeda aralocaspica,* which have independent evolutionary origins and anatomical forms of SC-C_4_. Taken together, the integrated structural, biochemical, and functional analyses presented here reveal a stepwise progression in the development of C_4_ type chlorenchyma cells. This study provides new insights into processes responsible for the specialized photosynthetic characteristics of these unique plants. The findings highlight the dramatic differences in development of single-cell C_4_ compared to sister Kranz-type *Suaeda* species, and they suggest diversity exists in how regulatory factors control the evolution of different forms of C_4_.

## Materials and methods

### Plant material

The SC-C_4_ species *Bienertia sinuspersici* Akhani and *Suaeda aralocaspica* (Bunge) Freitag & Schütze (syn.=*Borszczowia aralocaspica* Bunge) were used in this study. These are classified as C_4_ structural forms called Bienertioid and Borszczowoid, respectively (Edwards and Voznesenskaya, 2011). Seeds of *S. aralocaspica,* originally collected in Kazakhstan, were germinated on moist paper towels in Petri dishes for 1–2 d at 22°C. After the radical appeared, seeds were transferred to a soil mixture of one part potting soil, two parts sand, 0.25 part gypsum, 0.5 part Perlite, and 0.5 part clay. *B. sinuspersici* Akhani (seeds originally from Kuwait) was propagated from cuttings in rooting MS media and transferred to potting soil according to the protocol of Smith *et al.* (2009).

Plants were grown in a growth chamber (model GC-16; Enconair Ecological Chambers Inc., Winnipeg, Canada) under a 14/10h 25/18°C day/night cycle under mid-day PPFD of ~400 μmol quanta m^–2^ s^–1^, and 50% relative humidity for ~2 months. Plants were fertilized once a week with Peter’s Professional (20:20:20; Scotts Miracle-Gro Co., Marysville, OH, USA) and watered once a week with 150mM NaCl.

For microscopy and biochemical analyses, leaf samples were taken from vegetative branches on ~2 month old plants. Mature leaves of *B. sinuspersici* are 2.5–3cm in length, and *S. aralocaspica* 1.5–2cm in length; for studies on transitions along a longitudinal gradient young leaves 0.5–0.7cm long were used (see Supplementary Fig. S1, available at *JXB* online, for a general view of mature and young leaves).

Voucher specimens are available at the Marion Ownbey Herbarium, Washington State University: *Suaeda aralocaspica* (E. Voznesenskaya 22), April 2006, WS369790 and *Bienertia sinuspersici* (E. Voznesenskaya 85), May 2013, WS386421.

### Light and electron microscopy

Developmental studies were carried out on young expanding leaves and on mature leaves that were fully expanded. For structural studies, for each developmental stage sampled, three replicates were taken from three independent plants for each species (i.e. a total of nine samples for each species). Vegetative shoot apices with several leaf primordia (up to 0.3cm), and young leaves (0.5–0.7cm in length), were harvested and prepared for longitudinal and cross sectioning.

Sample preparation for light microscopy (LM) and transmission electron microscopy (TEM) was carried out according to Koteyeva *et al.* (2011). An Olympus BH-2 (Olympus Optical Co. Ltd) light microscope equipped with LM Digital Camera and Software (Jenoptik ProgRes Camera, C12plus, Jena, Germany) was used for observation and collection of images on LM level. Hitachi H-600 (Hitachi Scientific Instruments, Tokyo, Japan), and FEI Tecnai G2 (Field Emission Instruments Company, Hillsboro, OR, USA) equipped with Eagle FP 5271/82 4K HR200KV digital camera transmission electron microscopes were used for TEM studies.

Observations and image capture of vegetative shoot apices with the youngest primordia were obtained by scanning electron microscopy, using the low vacuum mode on an FEI SEM Quanta 200F (FEI Company, Field Emission Instruments, Hillsboro, OR, USA).

Observations of vascular development were obtained from leaves of different ages, from the youngest primordia (starting from ~0.3mm long) to fully expanded leaves (2.5–3cm for *B. sinuspersici* and 1.5–2cm for *S. aralocaspica*). These samples were cleared in 70% ethanol (v/v) until chlorophyll was removed, treated with 5% (w/v) NaOH overnight, and then rinsed three times in water. At least five vegetative shoot tips with different-aged leaves from two or three different plants were used. The leaves were mounted in water and examined under UV light (with DAPI filter) on a Fluorescence Microscope Leica DMFSA (Leica Microsystems Wetzlar GmbH, Germany) using autofluorescence of lignified tracheary elements of the xylem.

### *In situ* immunolocalization

Sample preparation and immunolocalization by LM and TEM was carried out on longitudinal sections of leaves 0.5–0.7cm long according to the procedures in Koteyeva *et al.* (2011). Antibodies used (all polyclonal raised in rabbit) were anti-spinach Rubisco (rbcL) IgG (courtesy of B. McFadden), and commercially available anti-maize PEPC IgG (Chemicon, Temecula, CA, USA). The density of labeling was determined by counting the gold particles on digital electron micrographs using the UTHSCSA image analysis program and calculating the number per unit area (μm^2^). Additional details are available in Supplementary Methods S1 available at *JXB* online.

### *In situ* localization of mRNAs encoding Rubisco large subunit protein

Sample preparation and *in situ* hybridization was carried out on longitudinal sections of leaves 0.5–0.7cm long according to the procedures in Koteyeva *et al.* (2011). Sense and antisense RNA probes for Rubisco *rbc*L were generated from pBlsl (which contains a 600-bp HindIII fragment from the central coding region of the amaranth *rbc*L gene) using a modification of procedures described in Wang *et al.* (1992). Sense and antisense transcripts were synthesized and labeled *in vitro* with Biotin-11-UTP (Roche) using T7 or T3 polymerase (Roche). Samples of young leaves were fixed in FAA (50% ethanol, 5% glacial acetic acid, 10% formalin) fixative at room temperature overnight. After ethanol and t-Butyl alcohol dehydration, samples were embedded in Paraplast Plus. The paraffin-embedded samples were sectioned (thickness 5–10 µm) using a rotary microtome; sections were mounted to poly-L-lysine-coated slides, dried, and stored at 4°C overnight. After deparaffinization by xylene, the sections were rehydrated through an ethanol series, incubated in 0.2M HCl for 20min at room temperature (RT), and rinsed in H_2_O. Slides were then incubated in 2×SSC (1×SSC is 0.15M NaCl, 0.15M sodium citrate) at 70°C for 30min and rinsed by H_2_O. Treatment of sections with proteinase K (1 µg ml^−1^ in TE: 100mM TRIS pH 8.0, 50mM EDTA) for 15min at 37°C was followed by a brief rinse in PBS (10mM phosphate buffer, 2.7mM KCl, 137mM NaCl, pH 7.4), blocking in glycine (2mg ml^–1^ in PBS) for 2min at RT, and then by fixation for 20min in 4% formaldehyde. The sections were incubated in 2×SSC for 10min at RT, and then placed into prehybridization medium [containing 1.25× *in situ* salts (10× salts: 3M NaCl, 0.1M Tris, 0.1M NaHPO_4_, 50mM EDTA, pH 6.8), 50% deionized formamide, 1mg ml^−1^ tRNA, 125mM DTT, 0.5mg ml^−1^ polyA] overnight at 50°C. The probes were first heated at 75°C for 30s and mixed with prehybridization medium with a final transcript concentration of 0.5 µg ml^–1^. Hybridizations with the Biotin-labelled transcripts were performed at 50°C overnight in a moist incubation chamber. The slides were then subjected to the following series of washes: prewarmed at 37°C in 2×SSC for 10min; 2×SSC for 1h at RT; 1×SSC for 1h at RT; 0.5×SSC for 30min at 42°C; 0.5×SSC for 30min at RT. Hybridized transcripts were detected by streptavidin–alkaline phosphate conjugate (NeutrAvidin; Pierce) using blocking buffer (100mM TRIS–HCl, pH 7.5, 150mM NaCl). The final detection step was carried out by nitroblue tetrazolium chloride and 5-bromo-4-chloro-3-indolyl phosphate (Sigma). Reactions were stopped by placing slides in 1×PBS and the sections were mounted in 50% glycerol in PBS. Observations and image capture were performed using an Olympus BH-2 light microscope equipped with LM Digital Camera and Software.

### Western blot analysis

Analyses were made to determine accumulation of photosynthetic enzymes in samples of young leaves 0.5–0.7cm long divided into three sections (base, middle and tip) compared to mature leaves. Western blots were performed using anti-*Amaranthus hypochondriacus* NAD-malic enzyme (NAD-ME) IgG against the 65 KDa α subunit (Long and Berry, 1996) (1:5000), anti-*Zea mays* PEPC IgG (1:100 000), anti-*Zea mays* pyruvate,Pi dikinase (PPDK) IgG (courtesy of T. Sugiyama) (1:5000), anti-*Amaranthus hypochondriacus* Rubisco SSU IgG (courtesy of J. Berry) (1:5000); or anti- *Spinacia oleracea* Rubisco rbcL IgG (courtesy of B. McFadden) (1:10 000) overnight at 4°C. For details see Supplementary Methods S1, and Supplementary Fig. S2 for a loading control with protein samples (10 µg) separated by 10% (w/v) SDS-PAGE.

### Mass spectrometric measurements

A membrane inlet mass spectrometer (DELTA V Plus; Thermo Scientific) connected to a closed leaf chamber via a membrane inlet was used to measure rates of isotopic CO_2_ exchange and CO_2_ compensation points (Γ), as described previously (Maxwell *et al.*, 1998; Walker and Cousins, 2013). Leaves were detached from different branches and placed into the chamber at ambient CO_2_ and O_2_ partial pressures (Supplementary Fig. S1C–F). For analysis with *B. sinuspersici*, 3–4 mature leaves or 16–18 young leaves (length ~0.5cm) were used; for analysis with *S. aralocaspica*, 5–8 mature leaves or 30–40 young leaves (length ~0.6cm) were used. The leaf chamber was flushed with mixture of nitrogen and corresponding oxygen partial pressure and then sealed. After sitting for 5min in the dark, the leaves were illuminated with 1000 µmol quanta m^–2^ s^–1^, with a chamber temperature of 25°C controlled by a circulating water bath. Net CO_2_ assimilation was followed in the sealed chamber by measuring the change in CO_2_ concentration until the Γ was reached (i.e. when the amount of CO_2_ assimilated by photosynthesis was balanced with the amount of CO_2_ released by respiration and photorespiration, Г was recorded). The response of Γ to changes in O_2_ was measured at 10%, 20% and 40% (at partial pressures of 0.092, 0.184 and 0.369mbar O_2_, respectively). The rate of dark respiration was calculated by measuring the amount of CO_2_ release in the dark. The zero set point was recorded, before and after each measure, by flushing the chamber with a corresponding CO_2_-free nitrogen and O_2_ mixture.

### Statistical analysis

Where indicated, standard errors were determined, and analysis of variance (ANOVA) was performed with Statistica 7.0 software (StatSoft, Inc.). Tukey’s HSD (honest significant difference) test were used to analyze differences between amounts of gold particles, intensities of bands in western blots and Г and dark respiration values at different stages of leaf development. All analyses were performed at the 95% significance level.

## Results

### Structure of shoot apex and early leaf development

Scanning electron microscopy and light microscopy were used to examine the morphology and anatomy of vegetative apical shoot meristems during active organogenesis of *B. sinuspersici* and *S. aralocaspica* in order to show the earliest stages of leaf initiation (Supplementary Fig. S2). The vegetative apices (apical meristems) of both species are very similar in shape and composition (Supplementary Fig. S2A–C, E, F). Leaf primordia are initiated alternately in both species forming the buds with more or less loosely packed leaves in *B. sinuspersici* and tightly packed leaves in *S. aralocaspica* (Supplementary Fig. S3 illustrates differences in branches and in leaf morphology of young versus mature leaves of the two species).

Observations on longitudinal sections of the youngest leaves (length 0.1–0.3mm) showed differences in the origin of chlorenchyma cells from the ground meristem. Periclinal divisions of subepidermal cells give rise to two layers of chlorenchyma in *B. sinuspersici*, versus a hypodermal and a chlorenchyma layer in *S. aralocaspica* (not shown).

**Fig. 1.**
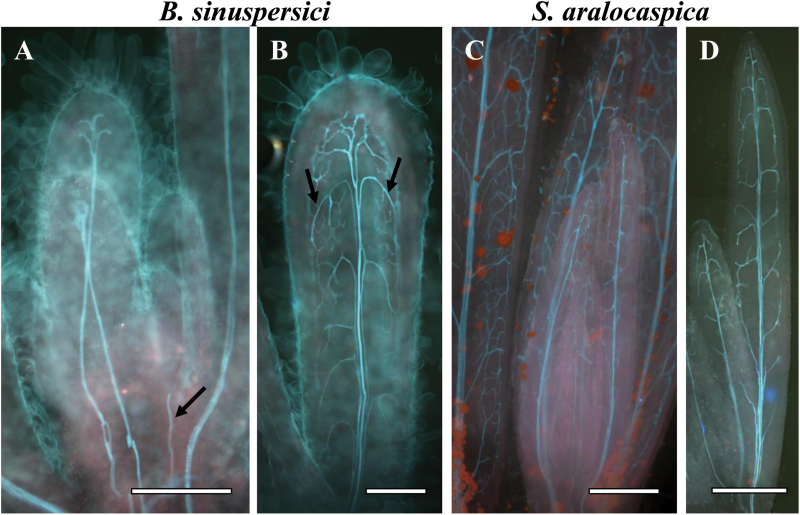
Cleared young leaves of *Bienertia sinuspersici* (A, B) and *Suaeda aralocaspica* (C, D) viewed under UV light at different stages of vein initiation. There is acropetal formation of the central vein towards the leaf tip (illustrated by arrow in panel A) and basipetal direction of lateral vascular vein development from the tip to base of the leaf (illustrated by arrows in panel B). Scale bars: 250 μm for A, B; 500 μm for C, D.

The development of vascular tissue was studied in young, cleared leaves of different lengths. The analysis showed that the central vein was formed acropetally from the base to tip in both species, beginning in leaves that were ~0.5mm long (a forming vein in *B. sinuspersici* is illustrated in Fig. 1A with an arrow). Peripheral veins were developed basipetally in both species (indicated by the label in Fig. 1B), starting from the tip of 0.7–1mm leaves with descending interconnecting loops (Fig. 1A, C). This was followed by dense reticulation that progressed along with continuing leaf growth (Fig. 1B, D).

### Chlorenchyma developmental gradient along the leaf: light and transmission electron microscopy

**Fig. 2.**
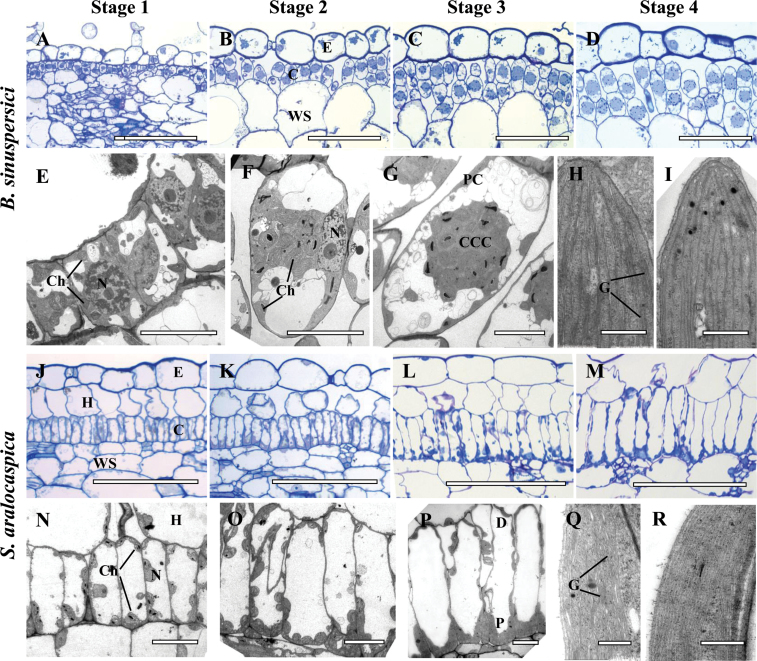
Light and electron microscopy of *Bienertia sinuspersici* (A–I) and *Suaeda aralocaspica* (J–R) with longitudinal sections of young leaves. The sections show a basipetal developmental gradient with gradual structural differentiation of SC-C_4_ chlorenchyma cells with four stages along the longitudinal gradient: Stage 1 (A, E, J, N), Stage 2 (B, F, K, O), Stage 3 (C, G, L, P) and Stage 4 (D, H, I, M, Q, R). Panels A–D from *B. sinuspersici* and J–M from *S. aralocaspica* are light microscopy micrographs of longitudinal sections showing the development of chlorenchyma cell lineages from the base (A, J) to the tip (D, M) of young leaves, with the direction of maturation from left to right. Panels E–G from *B. sinuspersici* and N–P from *S. aralocaspica*, are TEM micrographs showing internal structural development within a single chlorenchyma cell along the longitudinal gradient from the base (E, N) to the middle region (G, P) of a young leaf. Panels H, I, show the ultrastructure of chloroplasts in the central cytoplasmic compartment, CCC (panel H) and the periphery (panel I) within a single chlorenchyma cell at the tip of a young *B. sinuspersici* leaf. Panels Q, R show ultrastructure of chloroplasts within the proximal (Q) and distal (R) regions of chlorenchyma cell at the tip of a young *S. aralocaspica* leaf. E, epidermis; C, chlorenchyma; CCC, central cytoplasmic compartment; D, distal end; G, grana; H, hypoderm; N, nucleus; P, proximal end; WS, water storage. Scale bars: 100 μm for A–D and J–M; 10 μm for E–G and N–P; 0.5μm for H, I, Q, R. (This figure is available in color at *JXB* online.)

The structural differentiation of chlorenchyma cells was examined using longitudinal sections of young leaves (0.5–0.7cm long) that possess a basipetal developmental gradient with fully differentiated tissues at the tip (Fig. 2). Clear differences were observed in chlorenchyma cell differentiation between the tip and the base of leaves with a gradual developmental transition in structural specialization that was coupled with cell expansion. For comparison of the results the developmental progression was divided into four stages, with numbering 1 through 4 beginning at the basal region and towards the tip of the leaves (Fig. 2A–D and E–I for *B. sinuspersici*, J–M and N–R for *S. aralocaspica*).

The chlorenchyma cells near the leaf base (Stage 1) were found to be small and uniform. These cells undergo periclinal and anticlinal divisions in *B. sinuspersici* (Fig. 2A, E), and only anticlinal division in *S. aralocaspica* (Fig. 2J, N). The peripheral vascular tissues are immature at the leaf base, consisting of xylem elements in the process of differentiation, and undifferentiated phloem elements (not shown).

In *B. sinuspersici*, Stage 1 (at the leaf base) chlorenchyma cells have a dense cytoplasm, a centrally located large nucleus, and multiple small vacuoles. There are a few small chloroplasts, which are distributed throughout the cytoplasm (Fig. 2E). SC-C_4_ development initiates at Stage 2 (above the base towards the mid-section), where differentiation of the unique chlorenchyma cell morphology is first observed. At this stage, there is development of vacuoles at opposite poles of the cell, while chloroplasts and mitochondria continue to multiply and begin to aggregate at the center of the cell next to the nucleus, which is pressed against one side of the cell (Fig. 2F). In Stage 3 (mid-section of the leaf towards the tip, Fig. 2C, G) the following series of events in structural differentiation of SC-C_4_ were observed. First, is the formation of the CCC, which earlier in its development was connected to the PC on one side of the cell. The CCC is either attached to one of the radial cell walls and the nucleus (when visible it is usually adjacent to the CCC and the cell wall); or, as observed in Fig. 2G, the CCC is well delimited in the center of the cells. Examination by TEM shows that both PC and CCC chloroplasts have a similar structure, with small grana (not shown). In Stage 3, numerous mitochondria are selectively located within the CCC, but these are not fully differentiated. The peripheral vascular bundles at this stage have differentiated phloem and xylem elements (not shown). At Stage 4, near the leaf tip, development of the characteristic features of chlorenchyma cells that define SC-C_4_ in *B. sinuspersici* is complete (Fig. 2D). All of the CCCs are positioned at the central region of the cell, and are only connected with the PC by thin cytoplasmic channels. Structurally dimorphic chloroplasts that characterize this species are established in Stage 4, with well-developed grana in the CCC chloroplast (Fig. 2H), and a deficiency in grana in the peripheral chloroplasts (Fig. 2I). Also, in this stage the mitochondria have established a well-developed structure with clearly observable tubular cristae (not shown).

In *S. aralocaspica*, the basal Stage 1 chlorenchyma cells have a well-developed central vacuole that was observable from the earliest stages. The nucleus is located in the peripheral cytoplasm near the middle of the cell and chloroplasts are distributed along the cell periphery (Fig. 2J, N). Differentiation of these chlorenchyma cells, which is initiated in Stage 2, consists of cell elongation accompanied by the partitioning of organelles to distal and proximal ends, with multiplication of mitochondria and chloroplasts occurring at the proximal domain (Fig. 2K, O). At Stage 3, the cells were clearly more developmentally advanced, with an overall appearance that was mostly similar to the fully mature SC-C_4_ cells of Stage 4 (Fig. 2L, P). However, more detailed observations by TEM revealed that ultrastructural differentiation of chloroplasts and mitochondria is not completed at Stage 3 (not shown). Only in Stage 4 structurally distinct dimorphic chloroplasts were clearly present, with the proximal chloroplasts having well-developed grana (Fig. 2Q) compared to the distal chloroplasts (Fig. 2R). Also, mitochondria, which are localized to the proximal end of the cell, now show complete development, with a characteristic tubular structure (not shown).

### *In situ* hybridization of rbcL mRNA and immunocytochemistry of rbcL protein at different stages of leaf development

**Fig. 3.**
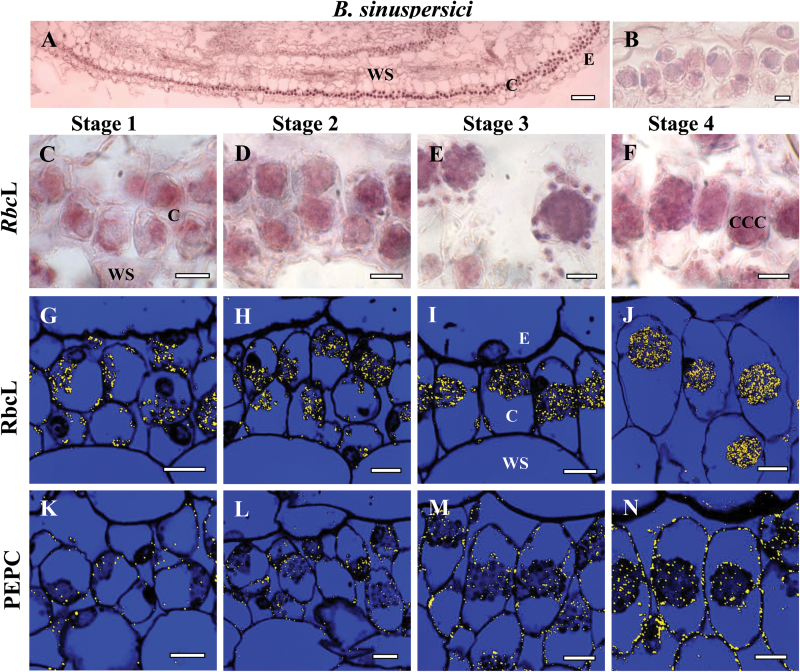
*In situ* hybridization of *rbc*L mRNA (A–F) and *in situ* immunolocalization of Rubisco rbcL (G–J) and PEPC (K–N) with longitudinal sections of young leaves from base to tip of *Bienertia sinuspersici* at four stages of development: Stage 1 (C, G, K), Stage 2 (D, H, L), Stage 3 (E, I, M) and Stage 4 (F, J, N). The dark purple signal indicates the specific hybridization to an antisense mRNA probe for *rbc*L mRNA (panels A, C–F). Yellow particles (panels G–N) indicate labeling with rbcL and PEPC antibodies. (A) Basipetal gradient of *rbc*L transcript accumulation from base (left) to tip (right). (B) Sense probe control showing the very low background staining that occurred in mRNA sense strand hybridization reactions. E, epidermis; CCC, central cytoplasmic compartment; C, chlorenchyma; WS, water storage. Scale bars: 100 μm for A; 10 μm for B–N.

**Fig. 4.**
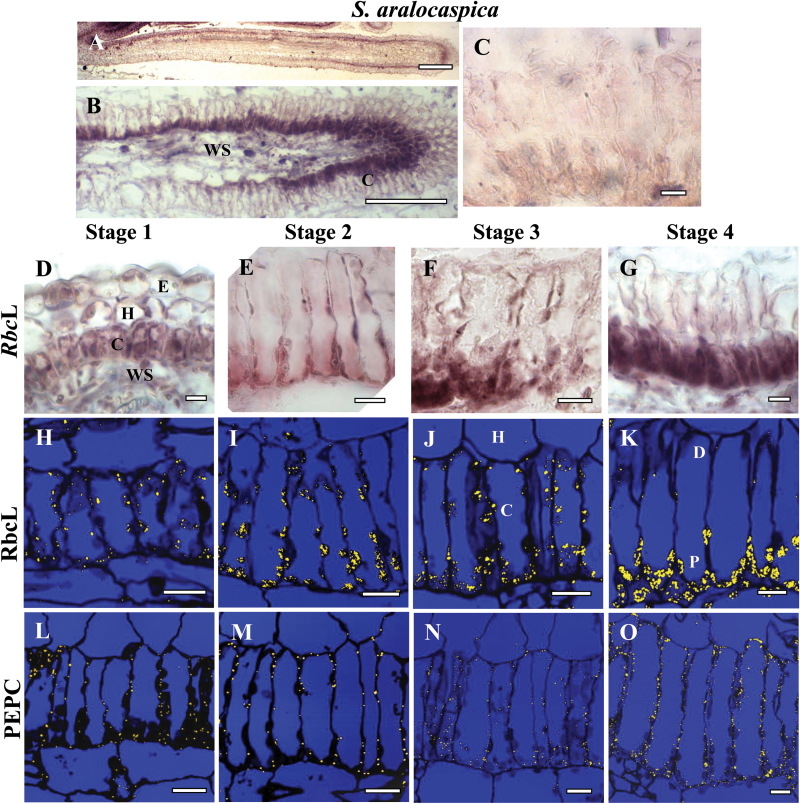
*In situ* hybridization of *rbc*L mRNA (A–G) and *in situ* immunolocalization of Rubisco rbcL (H–K) and PEPC (L–O) with longitudinal leaf sections from base to tip of *Suaeda aralocaspica* at four stages of development: Stage 1 (D, H, L), Stage 2 (E, I, M), Stage 3 (F, J, N) and Stage 4 (G, K, O). The dark purple signal indicates the specific hybridization to an antisense mRNA probe for *rbc*L mRNA (A, B, D–G). Yellow particles indicate peptide antibody labeling (H–O). (A, B) Basipetal gradient of *rbc*L transcript accumulation from base (left) to tip (right). (C) Sense probe control showing the very low background staining that occurs in mRNA sense strand hybridization reactions. E, epidermis; C, chlorenchyma; H, hypoderm; WS, water storage. Scale bars: 200 μm for A, 100 μm for B; 10 μm for C–O.

The temporal and spatial distribution of Rubisco *rbc*L mRNA was studied using *in situ* hybridization of biotin-11-UTP-labeled antisense RNA probes with longitudinal sections of young leaves (Fig. 3A, *B. sinuspersici*; Fig. 4A, *S. aralocaspica*). Specific hybridization was clearly visualized in chloroplasts as a dark purple color in *B. sinuspersici* (Fig. 3A, C–F) and *S. aralocaspica* (Fig. 4A, B, D–G). For a negative control, *rbc*L sense probes transcribed from the same plasmid in the opposite orientation showed little or no purple hybridization signal (Figs 3B, 4C). Antibody labeling of rbcL protein by immunolocalization with fluorescent confocal imaging appears in chloroplasts as yellow dots within the longitudinal sections (Figs 3G–J, 4H–K).

In *B. sinuspersici*, in the Stage 1 leaf region a clear hybridization signal for *rbc*L mRNA was observed within all of the chlorenchyma cell chloroplasts (Fig. 3C), as well as in the chloroplasts present in the epidermal and water storage cells. Also in Stage 1, confocal imaging revealed considerable labeling for rbcL, by immunolocalization, within all chloroplasts (Fig. 3G). In Stage 2, there was a similar pattern of hybridization for *rbc*L mRNA and immunolocalization of rbcL protein in all chloroplasts, as the developmental progression of cell elongation and position of organelles was beginning to occur (Fig. 3D, H). At Stage 3, as positioning of chloroplasts into the two separate domains was occurring, the hybridization signal for *rbc*L became more enhanced, with labeling observed in all of the chloroplasts (Fig. 3E). Also, at this stage immunolocalization indicated that all of the chloroplasts contained rbcL protein (Fig. 3I). At Stage 4, the mature chlorenchyma cells clearly showed selective localization of both *rbc*L mRNA and rbcL protein specifically within the CCC chloroplasts, with little or no signal observed in the peripheral chloroplasts (Fig. 3F, J).

In Stage 1 in *S. aralocaspica*, *in situ* hybridization and immunolocalization showed labeling for *rbc*L mRNA (Fig. 4D) and rbcL protein (Fig. 4H) within all of the chloroplasts. At Stage 2 and 3, while a clear increase in cytoplasmic volume and chloroplast number was observed within the proximal domain of chlorenchyma cells, all chloroplasts, in both the proximal and distal ends of the cell showed *rbc*L mRNA hybridization (Fig. 4E, F) and immunolocalization of rbcL protein (Fig. 4I, J). It was only in the most developmentally advanced Stage 4 towards the leaf tip that *rbc*L mRNA and rbcL protein became specifically confined to chloroplasts located within the proximal domain of the cell, with no labeling observed in the distal end chloroplasts (Fig. 4G, K).

**Fig. 5.**
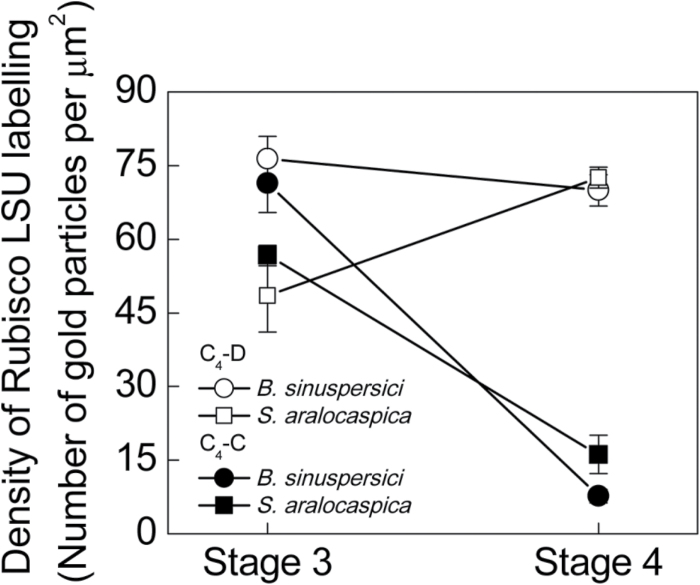
Quantitative graphical representation showing the density of immunolabeling for Rubisco rbcL in peripheral (●) versus CCC chloroplasts (○) in *Bienertia sinuspersici*, and in distal (■) versus proximal (□) chloroplasts of *Suaeda aralocaspica* at Stages 3 and 4 of development in young leaves. The *y* axis represents the number of gold particles per μm^2^ of chloroplast, and the *x* axis represents the developmental stages. 10–15 cell areas were used for counting in each cell type and for each stage of development.

For a more precise quantitative evaluation of rbcL protein in the different cell regions, visualization of immunogold antibody labeling by TEM was applied with cross-sections of leaves at Stages 3 and 4 of development. Counting of gold particles within different subcellular compartments at Stage 3 in both *B. sinuspersici* and *S. aralocaspica* confirmed a high level of labeling in chloroplasts located in both domains (Fig. 5). In the mature Stage 4 chlorenchyma cells, quantitative evaluation confirmed a dramatic change, with selective localization of immunogold labeling within the CCC chloroplasts of *B. sinuspersici*, and within the chloroplasts in the proximal domain of *S. aralocaspica*. Chloroplasts at the cell periphery in *B. sinuspersici* and in the distal compartment in cells of *S. aralocaspica* showed a dramatic drop in the density of gold particles as development progressed from Stage 3 to Stage 4 (9.3-fold in *B. sinuspersici* and 3.5-fold in *S. aralocaspica*) (Fig. 5). Interestingly, at the same time the density of labeling was declining in the distal chloroplasts in *S. aralocaspica* from Stage 3 to 4, there was a 1.5-fold increase in density in the proximal chloroplasts, while in *B. sinuspersici* maximum density of labeling for rbcL in the CCC occurred earlier, by Stage 3. In these young leaves there was very strong selective localization of rbcL to one chloroplast type at Stage 4; but some rbcL was detected by TEM in the distal chloroplasts of *S. aralocaspica* (10% of that in the proximal chloroplasts) and in the peripheral chloroplasts of *B. sinuspersici* (20% of that in the CCC chloroplasts) (Fig. 5).

### Immunocytochemistry of PEP carboxylase protein at different stages of leaf development

For both *B. sinuspersici* and *S. aralocaspica*, immunolabeling of PEPC at the leaf base (Stage 1, Figs 3K, 4L) and during the first stages of special chloroplast positioning (Stage 2, Figs 3L, 4M) was very weak in chlorenchyma cells, and absent in all other tissues. At Stage 3 the accumulation of PEPC was more prominent (Figs 3M, 4N) and at Stage 4 chlorenchyma cells showed the highest levels of cytosolic PEPC protein, with even distribution throughout the cytoplasm (Figs 3N, 4O).

### Western blot analysis of Rubisco and C_4_ pathway enzymes

**Fig. 6.**
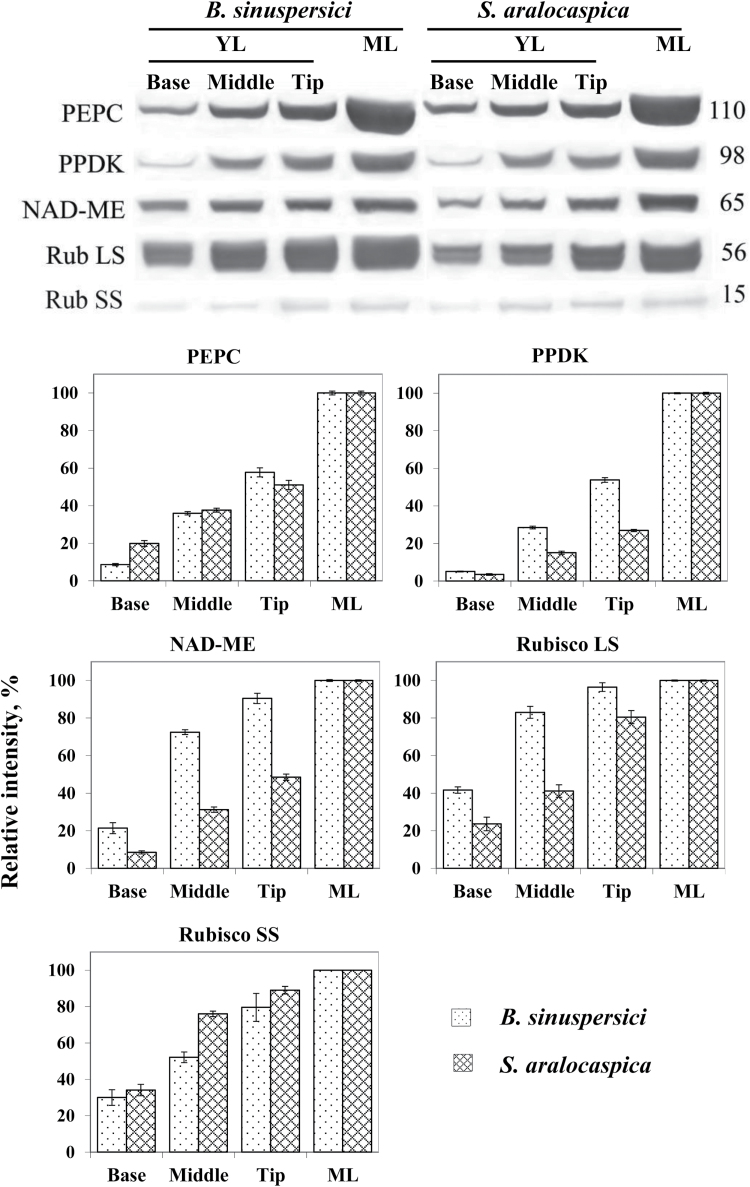
Western blot analysis showing accumulation of C_4_ enzymes, Rubisco rbcL and rbcS in protein extracts taken along the length of young leaf, and from mature leaves in *Bienertia sinuspersici* and *Suaeda aralocaspica*. Total soluble proteins were extracted from young leaves (0.5–0.7cm long) divided into three sections (base, middle, tip), and from mature fully expanded leaves. Blots were probed with antibodies raised against PEPC, PPDK, NAD-ME and Rubisco rbcL and rbcS, respectively. Top: Representative western blots showing detection of each protein with the antibody indicated. Numbers listed at the right indicate molecular mass in kilodaltons. Bottom panels: Quantitative representation of western blot data taking relative intensity of labeling of mature leaf as 100%. YL, young leaf; ML, mature leaf.

Accumulation of representative C_4_ pathway enzymes (PEPC, PPDK, NAD-ME) and Rubisco rbcL and rbcS, was analyzed by western blots during leaf development in *B. sinuspersici* and *S. aralocaspica*. Total soluble protein extracts prepared from the base (where most chlorenchyma cells are at Stage 1), middle (most chlorenchyma cells at Stages 2 and 3) and tip (chlorenchyma cells at Stage 4) of young leaves (length 0.5–0.7cm), and from mature leaves, were used for comparative developmental analysis (Fig. 6). All five proteins increased gradually as leaf development progressed from the base to the tip, with the highest levels of accumulation found in mature leaves. The rbcL content in *B. sinuspersici* at the base of the young leaf was 40% of that of mature leaves, increasing to 80% at the middle region of young leaves. In *S. aralocaspica*, rbcL was initially lower than in *B. sinuspersici*, with a more gradual increase in rbcL during development. The rbcS amount at the base of the young leaf was about 30% of mature leaf in both species and it increased towards the tip to 80 and 90% of mature leaf in *B. sinuspersici* and *S. aralocaspica*, respectively.

Among the three C_4_ cycle enzymes, levels of PPDK were found to be very low at the base of young leaves in both species (4–5% compared to mature leaves), with developmental increases lagging well behind those observed for Rubisco (Fig. 6). Both species showed initially low levels of PEPC at the base of the young leaves, with accumulation increasing along the gradient to maximum levels at the leaf tip, reaching up to ~50% of that in mature leaves. In *B. sinuspersici*, the levels of NAD-ME increased during development at a rate similar to rbcL, while its rate of increase in *S. aralocaspica* was notably slower.

### Analysis of function: CO_2_ compensation points and dark respiration

**Fig. 7.**
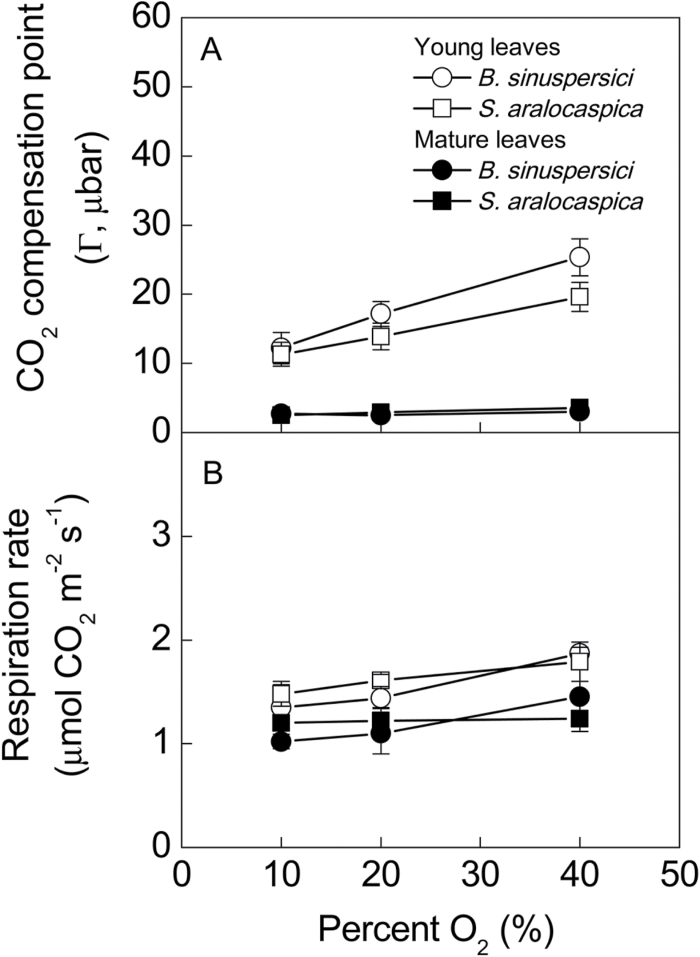
CO_2_ compensation points (Г) measured in the light, and rates of dark respiration at variable oxygen levels for mature (filled symbols) and young (open symbols) leaves of *Bienertia sinuspersici* (circles) and *Suaeda aralocaspica* (squares).

Mature leaves of *B. sinuspersici* and *S. aralocaspica* displayed low compensation point (Г) values (2.5 and 2.9 µbar) at 25ºC and current atmospheric levels of O_2_ (20%); and, these values were insensitive to changes in level of O_2_ from 10% to 20% to 40% (0.092, 0.184 and 0.369mbar O_2_) (Fig. 7A). However, in the young leaves of both species there was a linear increase in Г with increasing O_2_ from 10 to 40%. Additionally, at 25ºC and current ambient levels of O_2_ (20%), Г in young leaves (0.5–0.7cm) was ~7-fold higher than in mature *B. sinuspersici* leaves, and ~5-fold higher in *S. aralocaspica*. In comparing the two species, there was no significant difference (at *P*<0.05) between them for the value of Г in young leaves at 10 or 20% O_2_. However, Γ in *B. sinuspersici* appears to be more sensitive to increasing O_2_, and values at 40% O_2_ were significantly (at *P*<0.05) higher than in *S. aralocaspica.* With increasing O_2,_ there was no significant increase (at *P*<0.05) in the rate of dark respiration for either species. In both species, at a given level of O_2_, rates appeared to be slightly higher in young than in mature leaves; however, there was no significant difference at *P*<0.1 (Fig. 7B).

## Discussion

### Structural and biochemical transitions towards development of SC-C_4_

**Fig. 8.**
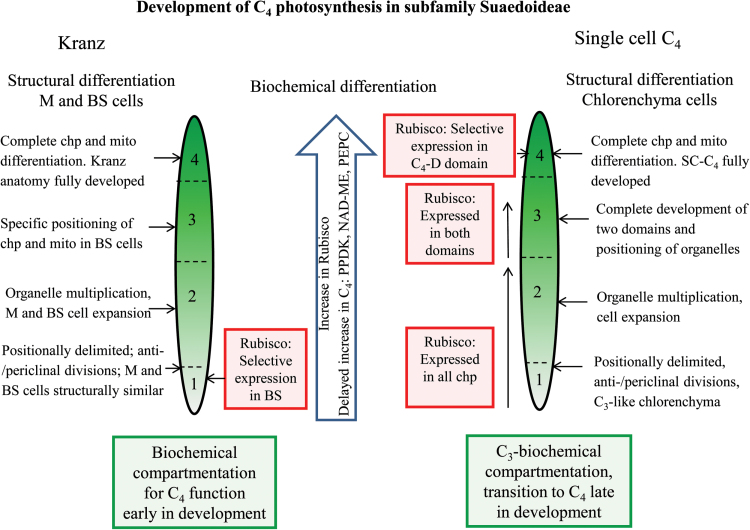
Illustrations of major differences during development of C_4_ photosynthesis from the basal region to the tip of young leaves in SC-C_4_ species versus Kranz-type C_4_ species in subfamily Suaedoideae. Progressive changes in differentiation in two types of SC-C_4_ species (*B. sinuspersici* and *S. aralocaspica*) are summarized from the current study, and compared with two structural forms of Kranz-type *Suaeda* species (*S. taxifolia* and *S. eltonica*) described previously (Koteyeva *et al.*, 2011). The numbers within the leaves refer to stages of chlorenchyma development along young leaves (length ~0.6cm). For comparison with SC-C_4_, the results with Kranz-type by Koteyeva *et al.* (2011) are summarized through four stages of development. The horizontal arrows point to specific defined changes that occur at each of the four stages. The vertical arrows indicate continual changes that occur along the longitudinal gradient. BS, bundle sheath; chp, chloroplasts; M, mesophyll; mito, mitochondria; SC-C_4_, single cell C_4_; C_4_-D domain, C_4_ cycle decarboxylation domain. (This figure is available in color at *JXB* online.)

The development, maintenance, and function of both forms of SC-C_4_ require extensive structural differentiation to form two intracellular domains that strictly partition different sets of biochemical functions within a single cell. *B. sinuspersici* and *S. aralocaspica* have independent evolutionary origins and possess structurally different forms of SC-C_4_ chlorenchyma cells. In both species, the chlorenchyma cells originate from the ground meristem; however, in *B. sinuspersici* periclinal divisions at the base of leaves give rise to two layers of chlorenchyma, while in *S. aralocaspica* periclinal divisions give rise to a single chlorenchyma layer and a layer of hypodermal cells. Previous analyses of mid-sections of leaves of different ages showed that chlorenchyma cells in young leaves have not developed the structural and biochemical properties that indicate transitions in development of SC-C_4_ chlorenchyma in *Bienertia* (Voznesenskaya *et al.*, 2005; Lara *et al.*, 2008; Park *et al.*, 2009) and *S. aralocaspica* (Voznesenskaya *et al.*, 2003a). In this current study, detailed structural and biochemical analyses of these species along longitudinal gradients of young leaves demonstrate that, despite having independent origins, different leaf anatomy and distinct chlorenchyma cell structures, the leaves of both species undergo very similar base-to-tip transitions to form C_4_ type chlorenchyma cells. Four stages of chlorenchyma development were recognized and defined with respect to structural and biochemical changes. Figure 8 indicates the progression of development of SC-C_4_ in both species, in comparison to related Kranz-type C_4_ species in subfamily Suaedoideae.

In Stage 1, at the base of the leaf in the meristematic zone, chlorenchyma cells of *B. sinuspersici* and *S. aralocaspica* contain a few, ultrastructurally monomorphic, chloroplasts. There is no evidence for development of the two cytoplasmic zones in either species. Analogous to Stage 1 in the SC-C_4_ species, Kranz-type C_4_ *Suaeda* species possess M and BS precursor cells that contain ultrastructurally identical organelles (both chloroplasts and mitochondria) (Koteyeva *et al.*, 2011).

At Stage 2 in both SC-C_4_ species and in Kranz-type *Suaeda* species, there is an increased expansion of photosynthetic cells. In *B. sinuspersici* the specialization of SC-C_4_ chlorenchyma cell structure continues with development of the CCC, together with enhanced chloroplast and mitochondria divisions around the nucleus. In *S. aralocaspica*, the chlorenchyma cells become increasingly elongated, accompanied by increasing cytoplasmic volume at the proximal end due to multiplication of chloroplasts and mitochondria. Kranz-type *Suaeda* species at this analogous stage of development are characterized by expansion of M cells with increase in vacuole volume and by multiplication of organelles in BS cells with initiation of their relocation to one side of the cell (Koteyeva *et al.*, 2011).

At Stage 3, two intracellular domains are morphologically evident in both SC-C_4_ species. These are precursors to the complete formation of C_4_-C and C_4_-D compartments. At this stage, chloroplasts within both domains still have a similar ultrastructure, but the mitochondria are now localized to the C_4_-D domain. At the analogous developmental stage in the *Suaeda* Kranz-type C_4_ species, the chloroplasts and mitochondria in the BS cells have relocated from their original position around the periphery of the cell to either a centripetal position adjacent to the vascular bundles, or to a centrifugal position, depending on species. The chloroplasts in the BS and M cells have similar ultrastructure (Koteyeva *et al.*, 2011), like chloroplasts in the two domains of Stage 3 SC-C_4_.

In both SC-C_4_ species, selective partitioning of chloroplasts and mitochondria into the two domains at Stage 3 is followed by later structural differentiation of these organelles in Stage 4. The C_4_-C domains contain chloroplasts with reduced grana formation, while chloroplasts in the C_4_-D domain have well developed grana. In NAD-ME type C_4_ species, including *B. sinuspersici* and *S. aralocaspica*, conversion of alanine and atmospheric CO_2_ to aspartate is considered to be the main flux in the carboxylation phase of the C_4_ pathway, a conversion that requires only ATP. The reduction in grana in the C_4_-C domain in NAD-ME type C_4_ species is considered to reflect a decreased requirement for PSII and NADPH production via linear electron flow (Hatch, 1987). Similarly, in the two structural forms of Kranz-type *Suaeda* species, complete differentiation to C_4_ chloroplasts and mitochondria also occurs during the final stage of leaf development (Koteyeva *et al.*, 2011). Thus, in all four distinct forms of C_4_ that occur in subfamily Suaedoideae, a final step in organelle differentiation is the specialized ultrastructural development of mitochondria and dimorphic chloroplasts. It is likely that signals regulating photochemical energy production between the two types of chloroplasts start to be transmitted after C_4_ function has distributed different demands for energy assimilation between the two domains.

Quantitative analyses of protein accumulation from western blots indicate a basipetal developmental gradient for both SC-C_4_ species, with levels of photosynthetic enzymes increasing along the length of the young leaves (Fig. 8). rbcL and rbcS proteins are relatively abundant in young cells at the leaf base, indicating substantial levels of Rubisco during early Stage 1 development. In comparison, at the leaf base there is little expression of the representative C_4_ cycle enzymes PEPC, NAD-ME and especially PPDK, which could be functionally rate-limiting for the developing C_4_ cycle at this early stage. Similarly, previous studies showed earlier accumulation of Rubisco (rbcL and rbcS) than the C_4_ enzymes in very young leaves, with greatly increased accumulation of Rubisco and C_4_ enzymes in mature leaves of *B. sinuspersici* and *B. cycloptera* (Voznesenskaya *et al.*, 2005; Lara *et al.*, 2008) and *S. aralocaspica* (Voznesenskaya *et al.*, 2003a). As with the SC-C_4_ species, photosynthetic enzyme levels in Kranz type *Suaeda* species increased along the leaf longitudinal gradient, with substantial Rubisco at the base of the leaf, and a lag in the accumulation of C_4_ enzymes, especially PPDK (Koteyeva *et al.*, 2011).

### Regulation of Rubisco expression in SC-C_4_

Mechanisms controlling the development of dimorphic chloroplasts in SC-C_4_ plants are not understood. Theories for selective targeting of nuclear encoded proteins to plastids in SC-C_4_ species (e.g. the small subunit of Rubisco, and PPDK) include selective import of required proteins into each plastid type, selective mRNA targeting from the nucleus to one domain and/or selective degradation of the protein following import (Offermann *et al.*, 2011). In a proteomics analysis of *B. sinuspersici* by Offermann *et al.* (2015), 35 nuclear-encoded proteins were found to be enriched in chloroplasts located to either the C_4_-C or C_4_-D domain. In fact, both groups of chloroplast proteins had predicted plastid transit peptides with classical physiochemical properties found in other species. These groups were comprised of proteins with different functional properties, including C_3_ and C_4_ carbon fixation pathways as well as certain proteins in light reactions.

The selective localization of Rubisco (consisting of the nuclear-encoded rbcS and chloroplast-encoded rbcL) for function within the C_4_-D domain is one of the most definitive biochemical features of SC-C_4_. Transient expression studies using constructs expressing a GFP fusion with the *B. sinuspersici* rbcS plastid transit peptide in *B. sinuspersici* SC-C_4_ mesophyll protoplasts provided no evidence for selective import within these cells (Rosnow *et al.*, 2014). In the current study, chloroplast-encoded Rubisco rbcL was used as an indicator of changes in gene expression that occurs during the differentiation of dimorphic chloroplast along the longitudinal gradient of SC-C_4_ development. *rbc*L mRNA, as well as its corresponding protein, was initially found within all chloroplasts of early chlorenchyma cells, both at the base (Stage 1) and middle (Stage 2, 3) regions of the young leaves. There were no quantitative differences in levels of Rubisco rbcL protein, mRNA or chloroplast structure that corresponded with the initial differentiation to form two domains in either of the SC-C_4_ species. Rather, selective partitioning of *rbc*L mRNA and its encoded protein to the C_4_-D domain only occurred later, during Stage 4 at the leaf tip. This localization was tightly coordinated with the establishment of chloroplast structural dimorphism. Thus, a ‘default’ C_3_-like pattern of *rbc*L mRNA and protein distribution appears to be maintained in chloroplasts during formation of the two cytoplasmic domains, with the more specialized SC-C_4_ distribution pattern becoming established only at the final developmental stage. Spatial and temporal accumulation patterns for Rubisco *rbc*L mRNA correlated with rbcL protein accumulation along the entire length of the longitudinal SC-C_4_ developmental gradient, indicating that transcript accumulation is a major determinant for Rubisco accumulation specifically within the C_4_-D domains of mature *B. sinuspersici* and *S. aralocaspica* leaves.

In contrast to SC-C_4_ species, two Kranz-type species of *Suaeda* (Koteyeva *et al.*, 2011) showed selective compartmentalization of *rbc*L mRNA and protein to BS chloroplasts much earlier, at Stage 1 (Fig. 8). In this case, cell-specific control of *rbc*L mRNA accumulation (by transcription or mRNA stability) occurs prior to the structural dimorphism and positioning of BS chloroplasts. In contrast, in the SC-C_4_ species, plastid-specific localization was observed only later, after development of the two cytoplasmic domains and chloroplast structural differentiation was complete. Taken together, these findings suggest different ontogenetic programs for development of C_4_ biochemistry have evolved independently in Kranz and SC-C_4_ species within the subfamily Suaedoideae.

Like the Kranz-type *Suaeda* species, early development of C_4_ has also been observed in some other Kranz-type C_4_ species. Positional control of Rubisco expression occurs very early in BS precursor cells in leaf primordia of maize (Nelson and Langdale, 1992), in BS cells of *Arundinella hirta* (Wakayama *et al.*, 2003), in BS cells at the base of young leaves in *Atriplex rosea* (Liu and Dengler, 1994; Dengler *et al.*, 1995), and in C_4_ species in family Cleomaceae (Koteyeva *et al.*, 2014). A similar pattern was shown also for three species of Cyperaceae (Soros and Dengler, 2001). However, in other Kranz-type species the pattern of Rubisco expression during leaf development was similar to that of SC-C_4_ species. *Amaranthus hypochondriacus* (Wang *et al.*, 1992, 1993) and *Salsola richteri* (Voznesenskaya *et al.* 2003b) show a prolonged C_3_-like distribution in M and BS chloroplasts in the early stages of leaf development.

Recently it was shown that the *rbc*L-specific mRNA binding protein RLSB has a role in post-transcriptional *rbc*L expression and possibly selective plastid-specific accumulation of Rubisco in several C_4_ species, including SC-C_4_ *B. sinuspersici* as well as the C_4_ dicots *Suaeda taxifolia* and *Flaveria bidentis*, and the C_4_ monocots *Zea mays* and *Setaria viridis* (Bowman *et al.*, 2013; Rosnow *et al.*, 2014). In *B. sinuspersici*, RLSB is co-localized with Rubisco specifically within the central compartment chloroplasts (Rosnow *et al.*, 2014), while in Kranz-type species it co-localizes with Rubisco only in BS chloroplasts. It has been proposed that RLSB and the differential redox status of the dimorphic chloroplasts might work together to selectively control synthesis of Rubisco in the C_4_-D domain in *B. sinuspersici* (Rosnow *et al.*, 2014). The established changes in *rbc*L mRNA and protein at different stages of leaf development demonstrated in this current study will serve as a framework for analyses currently in progress to determine how RLSB and other regulatory factors determine plastid-specific Rubisco accumulation in SC-C_4_ plant species.

### Functional analysis of young versus mature leaves

The leaves of *B. sinuspersici* and *S. aralocaspica* are too small to accommodate analysis of photosynthetic traits by traditional gas exchange methods from the individual defined regions along the longitudinal leaf gradient. Therefore, a functional analysis of photosynthesis on young compared to mature leaves was made by measuring the sensitivity of the CO_2_ compensation point (Г) to varying O_2_ concentrations using an inlet mass spectrometer. Typically at ~25°C, C_4_ species have a low Г between 1–5 µbar CO_2_, while in C_3_ plants the Г is 45–50 µbar CO_2_, and C_3_-C_4_ species have intermediate values in the range of 9–30 µbar CO_2_ (Krenzer *et al.*, 1975; Ku *et al.*, 1991; Vogan *et al.*, 2007; Voznesenskaya *et al.*, 2013). Increasing levels of O_2_ causes a linear increase in Γ in C_3_ plants (reflecting an increase in photorespiration), has little or no effect on Γ in C_4_ plants, but causes an intermediate, biphasic, response of Г in C_3_-C_4_ species (Ku *et al.*, 1991). In mature leaves of both SC-C_4_ species, Г was insensitive to increasing O_2_ levels up to 40%, with values characteristic of other C_4_ plants (2.5 to 3 µbar). However, in young leaves of both species, the Г values at 20% O_2_ were intermediate to those of C_3_ and C_4_ plants. Also, values of Γ increase as O_2_ was raised from 10 to 40% (~1.7-fold increase in *S. aralocaspica* and 2-fold increase in *B. sinuspersici*), which is indicative of an increase in rates of photorespiration (Brooks and Farquhar, 1985). Since dark-type respiration was not affected by increasing O_2_ from 10 to 20%, it is not considered to contribute to the O_2_-dependent increase in Γ. The sensitivity of Γ to increasing O_2_ in young but not mature leaves provides additional support for transitions from C_3,_ to intermediate, to a fully C_4_ state of photosynthesis along a longitudinal gradient in SC-C_4_ leaves.

C_3_ photosynthesis and photorespiration is expected at the base of young leaves where there is substantial Rubisco in all of the chloroplasts, with relatively low levels of other C_4_ enzymes. The two domains have not yet become established to support the C_4_ cycle, and are not yet capable of concentrating CO_2_ around Rubisco via dimorphic chloroplasts or for refixation of photorespired CO_2_ from mitochondria. However, at Stages 2 and 3, development of two separate domains is progressing with increased expression of both Rubisco and C_4_ enzymes. The partitioning between domains in the mid-section of young leaves is analogous to that of Kranz-like Type II C_3_-C_4_ intermediates. In C_3_-C_4_ intermediates Rubisco is localized in both M and BS chloroplasts. Intermediates reduce Γ and photorespiration by exclusive localization of glycine decarboxylase in BS mitochondria which allows photorespired CO_2_ to be partially refixed. Type II intermediates have in addition a partially functional C_4_ cycle (Edwards and Ku, 1987). In the mid-sections of young leaves of SC-C_4_ species, chloroplasts in both domains have Rubisco; however, the mitochondria, which are the sites of decarboxylation by glycine decarboxylase as a consequence of photorespiration and by NAD-ME in the C_4_ cycle, are localized only in the C_4_-D domain. Finally, towards the tip of young leaves, Rubisco is selectively compartmentalized within the C_4_-D domains of the chlorenchyma cells, and the SC-C_4_ anatomy and function have become fully developed with low photorespiration.

## Concluding remarks

Two very different ontogenetic patterns of development exist between the SC-C_4_ and C_4_ Kranz-type members of the subfamily Suaedoideae (family Chenopodiaceae). This includes differences in structural, biochemical and functional differentiation in SC-C_4_ species compared to the Kranz-type species. The SC-C_4_ leaves show a clear longitudinal progression from a C_3_-default to intermediate to a full C_4_ state, while C_4_ traits develop very early in the Kranz-type species. This implies major differences between these groups at the molecular level along the leaf longitudinal gradient for regulatory factors associated with photosynthetic gene expression (such as the selective compartmentalization of *rbc*L expression), as well as regulatory factors required for the development of structural features (such as differentiation of the unique SC-C_4_ cellular domains and partitioning of organelles versus differentiation of different cell types in Kranz-type C_4_ Suaedoideae). This strong difference in C_4_ development between SC-C_4_ and Kranz forms of C_4_ in subfamily Suaedoideae has wider implications for theories regarding the evolution of the C_4_ pathway.

In the SC-C_4_ species *B. sinuspersici* and *S. aralocaspica*, the acquisition of full C_4_ capability along the longitudinal leaf gradient is associated with a developmental shift from monomorphic to dimorphic chloroplasts, in coordination with plastid-specific partitioning of Rubisco *rbc*L mRNA and protein accumulation. Despite differences in anatomy and independent origin of these two species, the strong similarity in their development of C_4_ suggests comparative studies to identify regulatory factors controlling the development of SC-C_4_ will be of interest. Future studies will focus on the identification and characterization of regulatory factors and signaling processes responsible for SC-C_4_ development and how these processes differ and/or overlap with those responsible for C_4_ development in Kranz-type C_4_ species.

## Supplementary data

Supplementary data is available at *JXB* online.

Methods S1. Details of *in situ* immunolocalization and Western blot analysis.

Fig. S1. General views of the branches of *Bienertia sinuspersici* and *Suaeda aralocaspica* showing position of young leaves forming the vegetative buds, and excised mature and young leaves of *B. sinuspersici* and *S. aralocaspica* in the chamber for inlet mass-spectrometric measurements.

Fig. S2. Representative membranes stained with Ponceau S after transfer of proteins to nitrocellulose membrane and before immunoblotting.

Fig. S3. Structure of the vegetative shoot tip in *Bienertia sinuspersici* and *Suaeda aralocaspica*, consisting of the apical meristem and early leaf primordia.

Supplementary Data

## Abbreviations:

BS: bundle sheath
CCC: central cytoplasmic compartment
C_4_-C: C_4_ cycle carboxylation domain
C_4_-D: C_4_ cycle decarboxylation domain
M: mesophyll
NAD-ME: NAD-malic enzyme
PC: peripheral compartment
PEPC: phosphoenolpyruvate carboxylase
PPDK: pyruvate,Pi dikinase
SC-C_4_: single cell C_4_
Г: CO_2_ compensation point.
