# Supplementary material for: The unique structural and biochemical development of single cell C4 photosynthesis along longitudinal leaf gradients in Bienertia sinuspersici and Suaeda aralocaspica (Chenopodiaceae)
Source: J Exp Bot. 2016 Mar 8;67(9):2587–601. doi: 10.1093/jxb/erw082 (PMC4861011; doi:10.1093/jxb/erw082)
Supplement: Supplementary Data [file supp_67_9_2587__index.html]

The unique structural and biochemical development of single cell C4 photosynthesis along longitudinal leaf gradients in Bienertia sinuspersici and Suaeda aralocaspica (Chenopodiaceae) — The unique structural and biochemical development of single cell C4 photosynthesis along longitudinal leaf gradients in Bienertia sinuspersici and Suaeda aralocaspica (Chenopodiaceae) — Supplementary Data 

# The unique structural and biochemical development of single cell C4 photosynthesis along longitudinal leaf gradients in *Bienertia sinuspersici* and *Suaeda aralocaspica* (Chenopodiaceae)

## Supplementary Data

Data files

- supplementary\_methods\_figures\_S1\_S3.pdf - Supplementary Data
