# Supplementary material for: The unique structural and biochemical development of single cell C4 photosynthesis along longitudinal leaf gradients in Bienertia sinuspersici and Suaeda aralocaspica (Chenopodiaceae)
Source: J Exp Bot. 2016 Mar 8;67(9):2587–601. doi: 10.1093/jxb/erw082 (PMC4861011; doi:10.1093/jxb/erw082)
Supplement: Supplementary Data [file supp_erw082_supplementary_methods_figures_S1_S3.pdf]

The unique structural and biochemical development of single cell C_4 photosynthesis along longitudinal leaf gradients in *Bienertia sinuspersici* and *Suaeda aralocaspica* (Chenopodiaceae)

Nuria K. Koteyeva, Elena V. Voznesenskaya, James Berry, Asaph B. Cousins, and Gerald E. Edwards

Supplementary Methods S1

In situ immunolocalization

Young leaves from each species were fixed at 4°C in 2% (v/v) paraformaldehyde and 1.25% (v/v) glutaraldehyde in 0.05 M PIPES buffer, pH 7.2. The samples were dehydrated with a graded ethanol series and embedded in London Resin White (LR White, Electron Microscopy Sciences, Fort Washington, PA, USA) acrylic resin.

For light microscopy observations, longitudinal sections (0.8-1 μm thick), were placed onto gelatin coated slides and blocked for 1 h with TBST + BSA (10 mM Tris-HCl, 150 mM NaCl, 0.1% v/v Tween 20, 1% w/v bovine serum albumin, pH 7.2). The slides were then incubated for 3 h with the preimmune serum diluted in TBST + BSA (1:100), anti-Rubisco (1:500 dilution), or anti-PEPC (1:200 dilution). The slides were washed with TBST+ BSA and then treated for 1 h with protein A-gold (10 nm particles diluted 1:100 with TBST + BSA). After washing, the sections were exposed to a silver enhancement reagent for 20 min according to the manufacturer's directions (Amersham, Arlington Heights, IL, USA), stained with 0.5% (w/v) Safranin O, and imaged in a reflected/transmitted mode using a Zeiss Confocal LSM 510 Meta Laser Scanning Microscope (Carl Zeiss, Inc. Headquarters, Thornwood, NY, USA). The background labeling with pre-immune serum was very low, although some infrequent labeling occurred in areas where the sections were wrinkled due to trapping of antibodies/label (results not shown).

For observations at the TEM level, thin cross sections (~70 nm thick) on coated nickel grids were incubated for 1 h in TBST + BSA to block non-specific protein binding on the sections. The sections were then incubated for 3 h with the preimmune serum diluted in TBST + BSA anti-Rubisco (1:50). After washing with TBST + BSA, the sections were incubated for 1 h with Protein A-gold (10 nm) diluted 1:100 with TBST + BSA. The sections were washed sequentially with TBST + BSA, TBST, and distilled water, and then post-stained with a 1:4 dilution of 1% (w/v) potassium permanganate and 2% (w/v) uranyl acetate. Images were

collected using a FEI Tecnai G2 equipped with Eagle FP 5271/82 4K HR200KV digital camera transmission electron microscope.

Western blot analysis

The accumulation of photosynthetic enzymes during structural development was determined in samples from young leaves (0.5-0.7 cm) which were divided into three sections towards the base, middle and tip, and from fully expanded mature leaves (2.5-3 cm for *B. sinuspersici* and 1.5-2 cm for *S. aralocaspica*). Total soluble proteins were extracted from leaf samples by homogenizing 0.1-0.2 g of tissue in 0.1-0.2 ml of extraction buffer [100 mM Tris-HCl, pH 7.5, 10 mM $MgCl_2$, 1 mM EDTA, 15 mM (v/v) β-mercaptoethanol, 20 % (v/v) glycerol, and 1 mM phenylmethylsulfonyl fluoride]. Insoluble material was removed by centrifugation (5 min, 10,000g). The supernatant fraction was diluted 1:1 in 60 mM Tris-HCl, pH 7.5, 4% (w/v) SDS, 25 % (v/v) glycerol, 1% (v/v) β -mercaptoethanol, and 0.1% (w/v) bromphenol blue and boiled for 5 min for SDS-PAGE. Protein concentration was determined with an RCDC protein quantification kit (Bio-Rad), which tolerates detergents and reducing agents. Protein samples (10 µg) were separated by 10 % (w/v) SDS-PAGE, blotted onto nitrocellulose membrane and stained with 0.1% Ponceau S (w/v) in 1% acetic acid (v/v) as a loading control (Supplementary Fig. S1). After destaining in 1% acetic acid, proteins were probed with anti-*Amaranthus hypochondriacus* NAD-malic enzyme (NAD-ME) IgG against the 65 KDa α subunit (Long and Berry, 1996) (1:5,000), anti- *Zea mays* PEPC IgG (1:100,000), anti- *Zea mays* pyruvate,Pi dikinase (PPDK) IgG (courtesy of T. Sugiyama) (1:5,000), anti- *Amaranthus hypochondriacus* Rubisco SSU IgG (courtesy of J. Berry) (1:5,000); or anti- *Spinacia oleracea* Rubisco rbcL IgG (courtesy of B. McFadden) (1:10,000) overnight at 4°C. Goat anti-rabbit IgG-alkaline phosphatase conjugated secondary antibody (Bio-Rad) was used at a dilution of 1:10,000 for detection. Bound antibodies were visualized by developing the blots with 20 mM nitroblue tetrazolium and 75 mM 5-bromo-4-chloro-3-indolyl phosphate in detection buffer (100 mM Tris-HCl, pH 9.5, 100 mM NaCl, and 5 mM $MgCl_2$). The intensities of bands in western blots were quantified with an image analysis program (ImageJ 1.37v Wayne Rasband National Institutes of Health, USA) and expressed relative to level in the mature leaf which was set at 100%.

Supplementary Figures

The unique structural and biochemical development of single cell C_4 photosynthesis along longitudinal leaf gradients in *Bienertia sinuspersici* and *Suaeda aralocaspica* (Chenopodiaceae)

Nuria K. Koteyeva, Elena V. Voznesenskaya, James Berry, Asaph B. Cousins, and Gerald E. Edwards

Fig. S1. General views of the branches of *Bienertia sinuspersici* (A) and *Suaeda aralocaspica* (B) showing position of young leaves forming the vegetative buds. Excised mature (C, D) and young (E, F) leaves in the chamber for inlet mass-spectrometric measurements, *B. sinuspersici* (C, E) and *S. aralocaspica* (D, F). Scale bars: 0.5 cm for A – F.

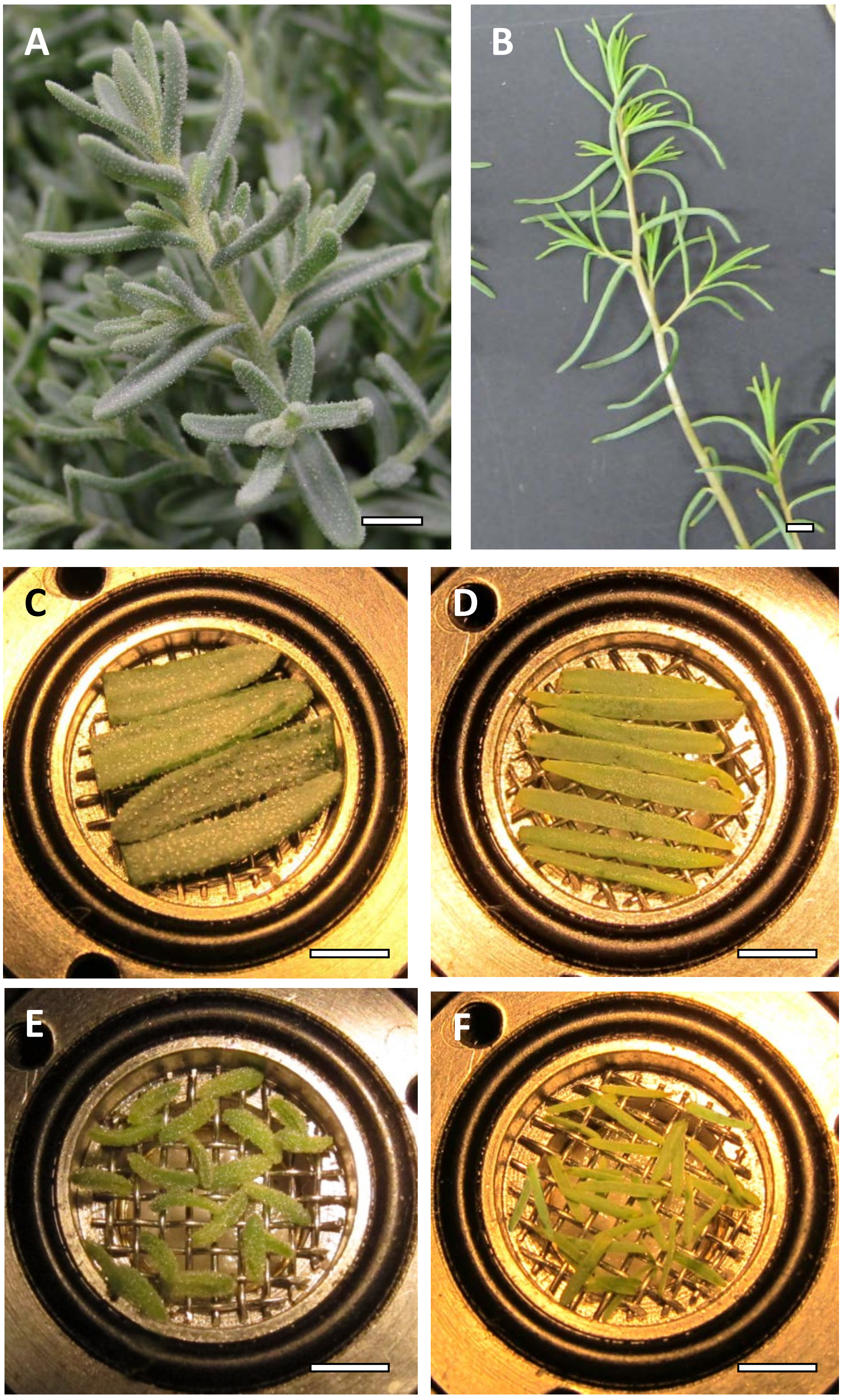

Fig. S2. Representative membranes stained with Ponceau S after proteins (10 µg per lane) transfer to nitrocellulose membrane and before immunoblotting.

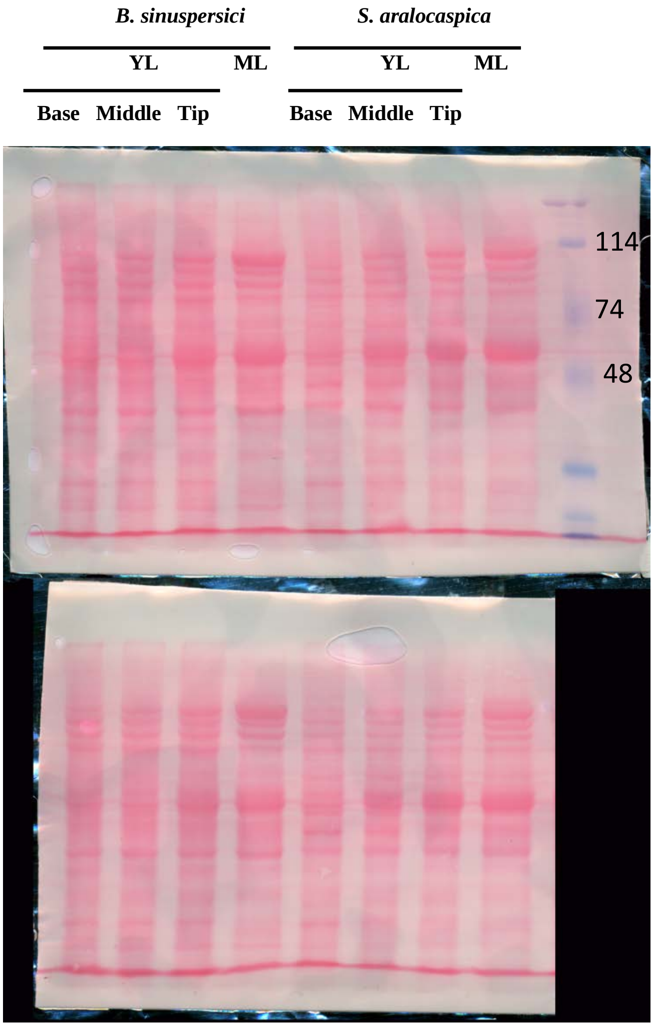

Fig. S3. Structure of the vegetative shoot tip, consisting of the apical meristem and early leaf primordia, in *Bienertia sinuspersici* (A-C) and *Suaeda aralocaspica* (D-F). A, D, Scanning electron microscopy of shoot tip with the positioning of young leaf primordia. B, E, Longitudinal section through the shoot tip showing early events of leaf initiation (axillary buds are illustrated by arrows). C, F, Light microscopy of the shoot apical meristem. C, corpus; LP, leaf primordium; T, tunica. Scale bars: 100 μm for A, B, D, E; 20 μm for C, F.

Fig. S3 shows morphology and anatomy of vegetative apical shoot meristems of *Bienertia sinuspersici* and *Suaeda aralocaspica* at the earliest stages of leaf development, during active organogenesis. The vegetative apices (apical meristems) of both species are dome shaped (Fig. S3 A-C, E, F), with a diameter at the base around 120 μm and a height of 100 μm. The apices consist of two layers of tunica, L1 and L2, where cells have mostly anticlinal divisions and 4-6 layers of central zone (corpus), where the cells divide in different planes (Fig. S3 C, F). Deeper in the tissue there are more heavily vacuolated cells, some of which will develop the core of the stem.

In *B. sinuspersici*, axillary buds are formed rather early during development; the 4th leaf pair (two pairs are visible in the image, and two pairs are in the opposite plane), located approximately 300 μm from the main apex, already has a rather large axillary meristem (Fig. S3 B). When growing under optimal conditions *B. sinuspersici* produces multiple stems and forms bushy plants due to intensive lateral branching (Fig. S1 A).

In contrast, *S. aralocaspica* is characterized by scarce branching. The buds are more compact in this species (Supplementary Fig. S1 B) having tightly packed leaves (Fig. S3 D). The emergence of young leaves from the external leaves, and their subsequent greening, occurs earlier in *B. sinuspersici* than in *S. aralocaspica.*

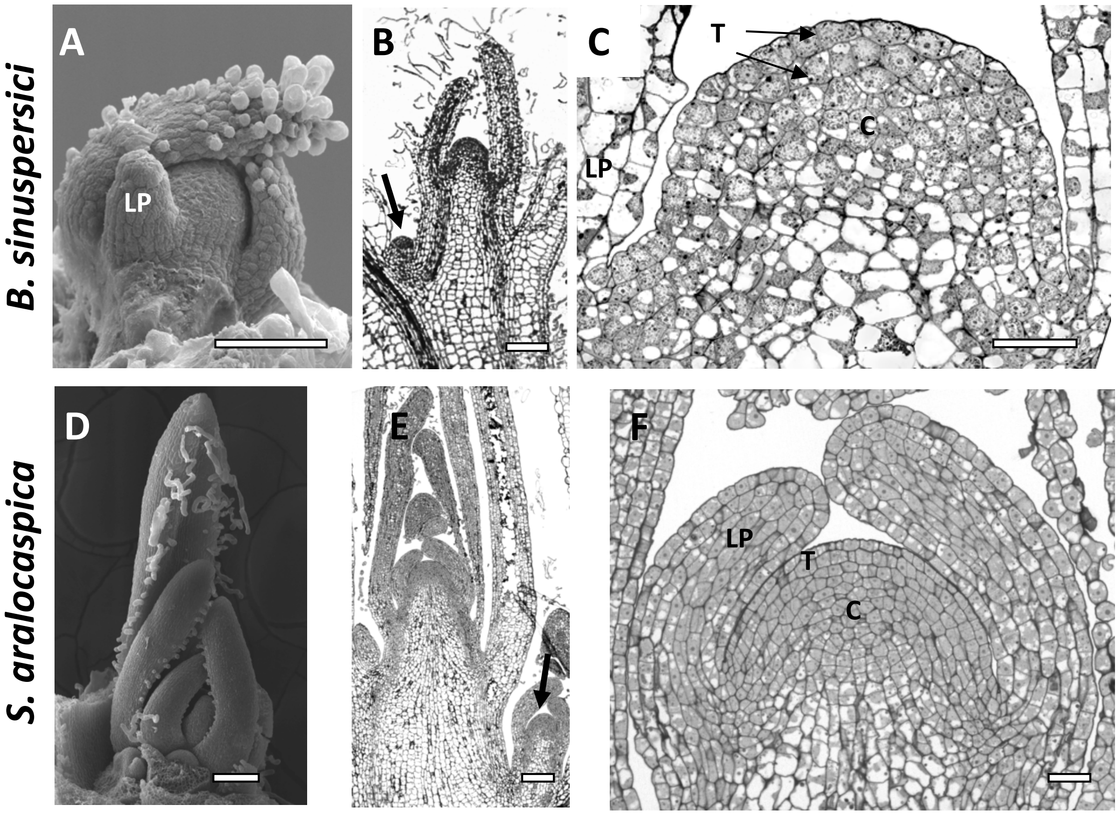
B. sinuspersici
S. aralocaspica
A
LP
B
C
T
C
LP
D
E
F
LP
T
C
